# AMMI and GGE biplot analysis of yield of different elite wheat line under terminal heat stress and irrigated environments

**DOI:** 10.1016/j.heliyon.2021.e07206

**Published:** 2021-06-03

**Authors:** Bishwas K.C., Mukti Ram Poudel, Dipendra Regmi

**Affiliations:** Department of Plant Breeding, Post Graduate Program, Institute of Agriculture and Animal Science, Tribhuvan University, Kirtipur, Kathmandu, Nepal

**Keywords:** Adaptability, Alpha-lattice, Biplot, Principal component

## Abstract

Wheat crop contributes to a major portion of the agriculture economy of Nepal. It is ranked as the third major cereal crop of the country even though, it faces terminal heat stress which speeds up the grain filling rate and shortens the filling period, causing reduction in grain weight, size, number and quality losses. We can minimize this loss through a genotypic selection of high-yielding lines by understanding the genotype-environment interaction. The objective of this research is to obtain a high yielding line with a stable performance across the environments. In order to do so, we conducted an experiment using eighteen elite wheat lines and two check varieties in alpha-lattice design with two replications in different environments viz. irrigated and terminal heat stress environment from November 2019 to April 2020. The analysis of variance revealed that genotype, environment and their interaction had a highly significant effect on the yield. Furthermore, the which-won–where model indicated specific adaptation of elite lines NL 1179, NL 1420, BL 4407, NL 1368 to the irrigated environment and Bhirkuti to the terminal heat-stressed environment. Similarly, the mean-versus-stability study indicated that elite lines BL 4407, NL 1368, BL 4919, NL 1350, and NL 1420 had above-average yield and higher stability whereas elite lines Gautam, NL 1412, NL 1376, NL 1387, NL 1404, and NL 1381 had below-average yield and lower stability. The ranking of elite lines biplot, PC1 explaining 73.6% and PC2 explaining 26.4% of the interaction effect, showed the rank of elite line, NL 1420 > NL 1368> NL 1350 > other lines, close to the ideal line. On the basis of the obtained results, we recommend NL 1420 with both the high yield and stability is suited across both the environments, while NL 1179 and Bhirkuti is adapted specifically for irrigated and terminal heat stress environment, respectively.

## 1 Introduction

The cultivation of wheat (*Triticum aestivum* L.) dates back to 10000 years ago when hunting and gathering society made a transition to agriculture. Wheat is an important human food crop and ranks among the top three cereals in the world because of its adaptability, nutritional value, and high yield potential (Shewry, 2009). Nepal has 35 improved cultivars, 540 landraces, and 10 wild relatives (Joshi et al., 2006). In Nepal, wheat occupies a major portion of the national economy and is used mainly for making bread and biscuits (Joshi et al., 2006). Furthermore, the grain along with stalk and chaff serves as important raw material for Nepalese industries, and stalk and chaff are also used in the form of mulch, construction material, and animal bedding (Oyewole, 2016).

In the past 10 years, Nepalese agriculture has seen a decrease in the cropping area of wheat and a very low increase in productivity. MoALD (2020) reports a decrease of 4% cropping area from 731131 ha in 2009/10 to 703992 ha in 2018/19. Similarly, in these ten years, the productivity increase has been very slow at the rate of just 0.102 ton/ha. The productivity at the start of the decade (2009/10) was 2.13 ton/ha and reached 2.85 ton/ha by the end of the decade (2018/19) (MoALD, 2020). If a side-by-side comparison between worldwide and nation wheat productivity of Nepal is to be done, the result does not look that promising for productivity in Nepal. The worldwide productivity of wheat was 3.32 ton/ha in 2015, 3.42 ton/ha in 2016, 3.54 ton/ha in 2017 and 3.43 ton/ha in 2018 whereas Nepal had productivity of wheat 2.59 ton/ha in 2015, 2.33 ton/ha in 2016, 2.55 ton/ha in 2017 and 2.76 ton/ha in 2018 (FAOSTAT, 2020). As a consequence of the low productivity, Nepal has been witnessing a gradual annual increase in wheat imports. Nepal's wheat import was 103705 tons in 2015, 217105 tons in 2016, 199626 tons in 2017, and 107467 tons in 2018 (FAOSTAT, 2020). If these imports are to be minimized, we can either increase productivity or increase the land cultivated under wheat, however the second option seems less feasible. Thus, the focus should be given to increase productivity by breaking the yield barrier through genetics and breeding tools, and mitigation of biotic and abiotic stress to wheat production (Chatrath et al., 2007). Fischer et al. (2014) calculated a yield gap of 26–69% for developing countries like Nepal. Research on breeding and suitable intensification in order to bridge this yield gap of wheat carries a huge potential.

Wheat is most suited to the temperate climate and high temperatures can negatively impact its yield (Olabanji et al., 2007, cited in Oyewole, 2016). The temperature requirement varies along with the plant growth stages (Oyewole, 2016) such as optimum range of temperature for growth during sowing is 16 °C–22 °C, for anthesis and grain filling is 12 °C–22 °C (Farooq et al., 2011) while during the period of ripening is 21 °C–25 °C (Flato et al., 2013). Beyond these ranges, the growth and yield of wheat are affected severely, hereby the situation of global climate change and temperature rise is a major risk in the wheat production system. This is because wheat when gets exposed to a temperature above 24 °C during anthesis and grain filling undergoes terminal heat stress causing yield reduction and the reduction increases with a longer exposure period (Prasad and Djanaguiraman, 2014). Global change in mean temperature will probably be in the range of (0.3–0.7) °C from 2016 to 2035 and is likely to change the agronomic practice developed over years (IPCC, 2014). This gradual increment in mean temperature is shortening the wheat growing season (Bita and Gerats, 2013). Together with this, erratic rainfall pattern has made it necessary to develop strategies which moderate the effect of several biotic and abiotic stress, and allows coping up with climate change effects (Buttar et al., 2012). Among all these stress, high-temperature stress during reproductive development is termed terminal heat stress (Suryavanshi and Buttar, 2016).

A significant part of the South Asian Region is under terminal heat stress including Nepal (Joshi et al., 2006). Heat stress causes multiple effects in wheat farming which include physiological effect (mainly chlorophyll deterioration and decreased leaf water), biochemical effect (especially reduced photochemical efficiency and stress metabolites accumulation), effect on growth and development (reduced growth duration, lower number of leaf and tiller formation), all of which leads to yield reduction (from quality to size, to crop stand and seed development) (Akther and Islam, 2017). An increase in temperature by (1–2) °C reduces grain mass which is mainly due to two reasons: accelerated grain growth rate and shortened grain filling period in wheat (Nahar et al., 2010). Findings also reveal a decrease of thousand grain weight by around 67.3% (Joshi et al., 2016) and yield loss in the range of 25%–35% (Bhatta et al., 2008) because of heat stress. In 2005, the grain yield decrease because of heat stress was 32 kg/ha (Tripathi et al., 2005) which climbed up to 1534 kg/ha (Poudel et al., 2020a) in 2020. Hence, it can be concluded that heat stress decreases grain weight (Pradhan and Prasad, 2015) and results in yield loss (Grant et al., 2011). Also, heat stress is a major predictor of wheat yield globally (Zampieri et al., 2017) and in Nepal, along with this, it also has a severe synergic effect with drought to decrease wheat yield drastically towards the end of the growing season (Pradhan et al., 2012).

The simplest and common solution for heat stress is the production of a new cultivar through genotype selection that can give a stable yield in adverse environments (Chapman et al., 2012; Nezhadahmadi et al., 2013). Although several improved varieties with high grain yield and stress tolerance have been developed despite that, problems exist in improving the productivity and profitability of wheat farming. Thus, there is a particular need to gain heat tolerance and develop heat-tolerant new germplasm through wheat breeding (Bhatta et al., 2008). The genotype selection depends on the understanding of the interaction among the genotypes, environment, and crop management practices which can be characterized using statistical methods (Jat et al., 2017). The variety with the highest average yield in all the test environment alone cannot be used for recommendation to the farmers; analysis of the stability of variety in the environment that is G × E interaction and physiological basis are also to be studied (Vergas et al., 2001; Thamson and Philips, 2006). Thus, stability analysis can be an effective tool to select genotype for heat tolerance.

The genotype with a low fluctuation of yield in stress and non-stress environment is stable and suitable for selection. For this, studies needs to focus on the interaction of genotypes with the environment and environmental stress such as heat (Hamada et al., 2007). This study is conducted to observe the yield stability of genotypes under heat stress and non-stressed (irrigated) environments.

## 2 Methodology

### 2.1 Field experimentation

The field experiment was conducted in two different environments viz. irrigated and terminal heat stress. The field in irrigated condition was sown in the last week of November to provide normal temperature to wheat crop in the reproductive and ripening stage. In the field of terminal heat stress, sowing was delayed by a month (the last week of December) to provide higher temperature to wheat crop in the reproductive and ripening stage which causes heat stress (Poudel et al., 2020b).

**Figure 1:**
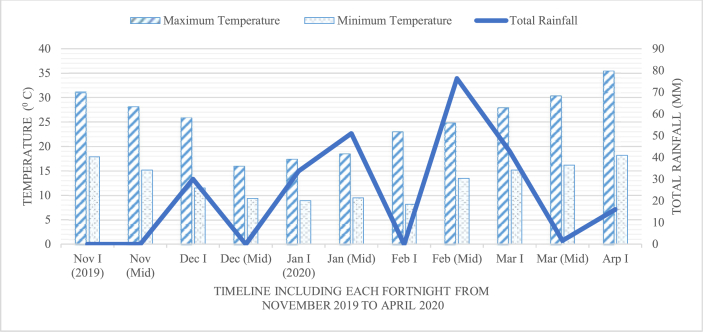
Maximum and minimum temperature; and the total rainfall during November 2019 to April 2020 in the experiment filed.

The record of maximum and minimum temperatures, and the total rainfall during each fortnight was obtained from National Wheat Research Program, Bhairahawa which is presented in Figure 1.

### 2.2 Soil properties

Soil samples obtained after land preparation was air-dried and well-grinded to sieve through a 2 mm sieve. Then soil characteristics analyzed in IAAS Soil Laboratory were noted down in Table 1.

**Table 1:** Soil properties of experiment field after land preparation.

Soil properties	Descriptions
Soil type	Clay loam
NPK content	0.47 kg per ha (high) Nitrogen,
	185 kg per ha (high) Phosphorus,
	122.5 kg per ha Potassium
Organic matter content	3.5%
Soil p^H^	5.3 (acidic)

The study of all these soil properties confirms growth and development of wheat in normal condition without any stress and nutrient deficiencies which may affect the yield.

### 2.3 Plant materials

The research is conducted with 20 wheat genotypes collected from National Wheat Research Program, Bhairahawa which includes 15 Nepal Lines (NL), 3 Bhairahawa lines (BL), and two commercial varieties Gautam and Bhirkuti as check varieties. The complete set of genotypes with their entry name is given in Table 2.

**Table 2:** List of elite wheat line with their origin, entry number as treatment.

Entry no.	Name of elite lines	Origin	Treatment
1.	Gautam	Nepal	T_1_
2.	BL 4669	Nepal	T_2_
3.	NL1412	CIMMYT, Mexico	T_3_
4.	BL 4407	Nepal	T_4_
5.	NL 1368	CIMMYT, Mexico	T_5_
6.	NL 1417	CIMMYT, Mexico	T_6_
7.	Bhrikuti	CIMMYT, Mexico	T_7_
8.	BL 4919	Nepal	T_8_
9.	NL 1376	CIMMYT, Mexico	T_9_
10.	NL 1387	CIMMYT, Mexico	T_10_
11.	NL 1179	CIMMYT, Mexico	T_11_
12.	NL 1369	CIMMYT, Mexico	T_12_
13.	NL 1350	CIMMYT, Mexico	T_13_
14.	NL 1420	CIMMYT, Mexico	T_14_
15.	NL 1384	CIMMYT, Mexico	T_15_
16.	NL 1346	CIMMYT, Mexico	T_16_
17.	NL 1404	CIMMYT, Mexico	T_17_
18.	NL 1413	CIMMYT, Mexico	T_18_
19.	NL 1386	CIMMYT, Mexico	T_19_
20.	NL 1381	CIMMYT, Mexico	T_20_

### 2.4 Experimental design and layout

The details of the experiment are presented in Table 3.

**Table 3:** Details of experimental design and layout.

Design	Alpha Lattice Design
Environments	Irrigated environment and heat-stressed environment (2 environments)
Treatment Details	20 treatments in 5 blocks each consisting of 4 treatments (in 4 plots)
Distance between any two blocks	1 m
Distance between plots within a block	0.5 m
Plot Area	10 m^2^,
Dimension	2.5 m × 4 m,
Sowing method	Continuous in a line
Number of rows	10 rows
Row – row distance	25 cm
Number of Replication (r)	2
Number of Blocks (b)	10
Number of blocks per replication (s)	5
Number of treatments per block (k)	4

#### 2.4.1 Terminal heat stress environment and irrigated environments

The 1-month delay sowing was performed to achieve high temperature during the anthesis and grain filling period of the plants under which the plant faces terminal heat stress. The normal date of sowing was performed for the irrigated environment (Poudel et al., 2020b).

### 2.5 Crop growth and management

The agronomic practices followed during the experiment are presented in Table 4:

**Table 4:** Details of crop growth and management in the experiment.

Tillage	Ploughing followed by harrowing 1 week prior sowing; harrowing and leveling at sowing.
Fertilization	
Farmyard Manure	5 ton per ha
Recommended dose	NPK 100: 50: 25 kg per ha.
Terminal heat stress	Full dose at land preparation.
Irrigated	Half nitrogen and full dose P, K at land Preparation Remaining half nitrogen at first irrigation.
Irrigation	5 times each at CRI, Heading, Flowering, Milking, and Soft dough stage of the wheat plant.
Weeding	Manually at heading stage.
Harvesting	Manually using sickles when all maturity indices were complete.
Threshing	Manually using sticks.

Sample from 1m^2^ was kept separate from each plot for data collection of yield and related yield attributes.

### 2.6 Statistical analysis

MS Office 2013 was used data entry and processing. The AMMI model with GGE bi-plots were used for analyzing the yield stability of elite lines in the heat stress and irrigated environment using GEAR (version 4.0, CIMMYT, Mexico).

Additive Main Effect and Multiplicative Interaction (AMMI) model was used for the mean of the yield of the 20 elite wheat lines from both the environments using GEAR software. The AMMI model equation is:

(1) $$Y_{ij}=\mu +\alpha_{i}+\beta_{j}+\sum_{n=0}^{N}\lambda_{n}\gamma_{in}\delta_{jn}\;+\theta_{ij}+\varepsilon_{ij}$$

where: Y_ij_ = the mean yield of elite line i in environment j, μ = the grand mean of the yield, α_i_= the deviation of the elite lines mean from the grand mean, β_j_ = the deviation of the environment mean from the grand mean, λ_n_ = the singular value for the PCA; n, N = the number of PCA axis retained in the model, $\gamma_{in}$ = the PCA score of an elite line for PCA axis n, δ_jn_ = the environmental PCA score for PCA axis n, θ_ij_ = the AMMI residual and $\varepsilon_{ij}$= the residuals. The degrees of freedom (DF) for the PCA axis were calculated based on the following method (Zobel et al., 1988).

(2) DF=G+E−1−2n

where: G = the number of elite lines, E = the number of environments, and n = the n^th^ axis of PCA.

The Genotype main effect plus Genotype by Environment interaction (GGE) biplot used principal component comprised of a set of elite lines scores multiplied by environment scores which gives a two-dimensional biplot (Ding et al., 2008) and simultaneous study of the genotype plus genotype-environment interaction was performed.

## 3 Results and discussion

### 3.1 AMMI model analysis

The result of the analysis of variance of the AMMI model revealed that grain yield is significantly (p < 0.001) affected by environment, genotype, and genotype-environment interaction, which explained 75.66 %, 17.25%, and 7.08 % of the occurred variation, respectively. Furthermore, it showed two PC with a highly significant differences (p < 0.001) and first interaction principal component of AMMI explaining 100% of the genotype-environment interaction with 36 degree of freedom (df) that is 19 df of PC1 and 17 df of PC2 as shown in Table 5.

**Table 5:** The analysis of variance of grain yield using AMMI models.

	DF	SS	MS	F-value	PROB(F)	% explained	% accumulated
ENV	1	47244306	47244306	1516.25∗∗∗	0	75.66	75.66
GEN	19	10773431	567022.7	18.2∗∗∗	0	17.25	92.92
ENV∗GEN	19	4423810	232832.1	7.47∗∗∗	0	7.08	100
PC1	19	4316190	227167.9	7.61∗∗∗	0	100	100
PC2	17	0	0	0	1	0	100
Residuals	40	1246350	31158.75	NA	NA	0	0

ENV – Environment, GEN – Genotype (elite wheat lines) PC – Principal Component of AMMI, DF – Degree of Freedom, SS – Sum of Square, MS – Mean Sum of Squares, '∗∗∗' - significant at p-value < 0.001.

AMMI model used for an accurate yield estimates (Zobel et al., 1988), summarizes the relationship of genotype and environment (Crossa, 1990), and provides a base of better use for other models (Gauch, 1988). AMMI revealed major part of the variation in yield is explained by environment which indicates environments were diverse. This finding is similar to Mohammadi et al. (2017), Ngailo et al. (2019), and Bhardwaj et al. (2020).

The AMMI biplot has the main effect as grain yield in the abscissa and the PC1 as the ordinate where the genotypes or environment which lies on the same vertical line have the same yield and which lies on the same horizontal line have the same interaction pattern. Also, the vectors of genotypes that have PC1 close to the origin (zero) have general adaptability whereas the vectors with larger PC1 are specifically adapted to an environment.

**Figure 2:**
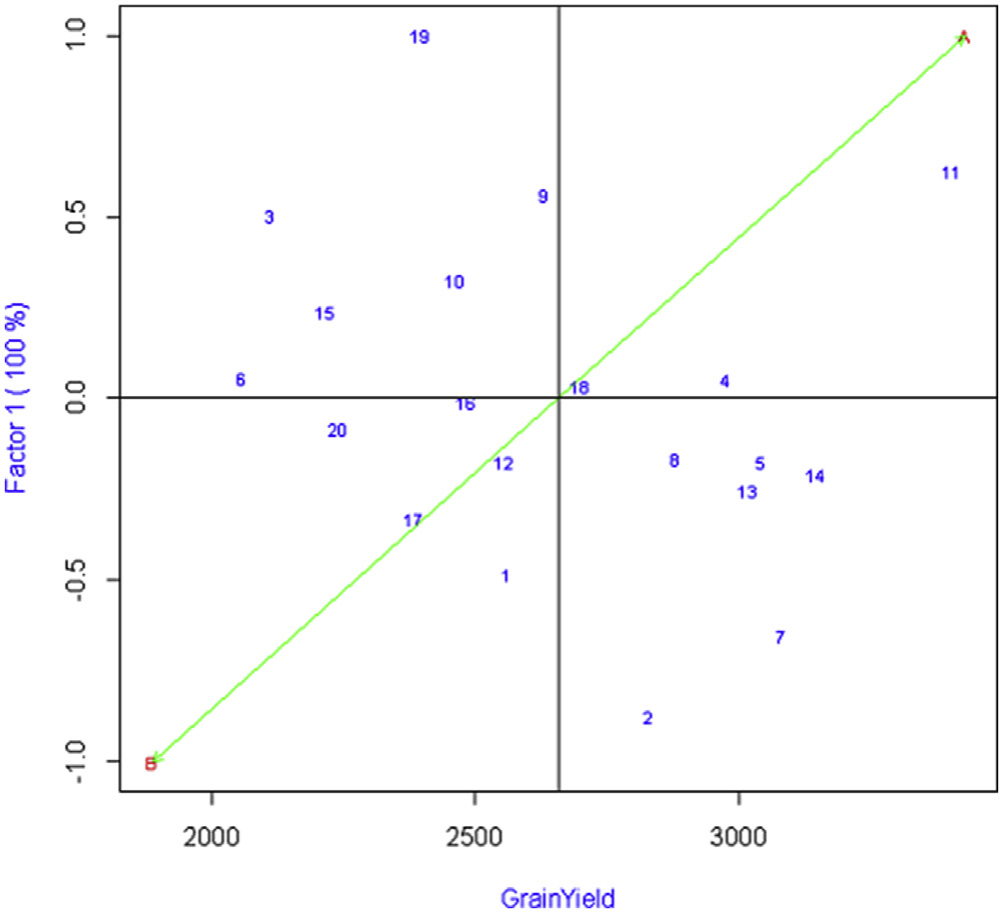
AMMI biplot PC 1 versus grain yield of 20 elite wheat lines in terminal heat stress and irrigated environments.

In the AMMI biplot, as shown in Figure 2, the genotypes that cluster together behave similarly across the environments. The elite wheat lines: NL 1368, BL 4919, NL 1350, NL 1420 are cluster close which performs similarly in both terminal heat stress and irrigated environment. The heat-stressed environment (B) has a lower than average yield and irrigated environment (A) have a higher than average yield. The elite wheat NL 1413 is the most stable among the tested line and BL 4407, BL 4919, NL 1387, NL 1369, NL 1346 are relatively stable lines in yield that are broadly adapted lines. The elite wheat BL 4669, NL 1412, NL 1417, Bhrikuti, NL 1179, NL 1386 are relatively unstable in yield because these lines are far from the origin and can be specifically adapted to an environment. Especially, NL 1179 are specifically adapted to irrigated environment, and Gautam, NL 1404, NL 1381 are specifically adapted to terminal heat-stressed environment.

Similarly, the PC 1 and PC 2 scores are reported as the representation of the stability of the lines across the environment that is the lines with the least PC scores have high stability and vice-versa. According to the PC1 score, BL 4669 with a score of -0.873 is the most stable followed by Bhrikuti, Gautam with a score of -0.653, -0.481 respectively while the PC2 score shows NL 1369, NL 1386, NL 1376 with a score of -2.28 × 10^−9^, -1.41 × 10^−9^, -7.92 × 10^−10^ respectively are the most stable lines with regards to yield across both the test environments as shown in the Table 6.

**Table 6:** Interaction principal component of AMMI (PC 1 and 2) with a yield of 20 test elite wheat lines.

	NAME	Yield	PC1	PC2
1	1(Gautam)	2560.5	-0.481	3.46 × 10^−09^
2	10 (NL 1387)	2463.25	0.324	-4.58 × 10^−10^
3	11 (NL 1179)	3405.25	0.627	9.24 × 10^−09^
4	12 (NL 1369)	2555	-0.174	-2.28 × 10^−09^
5	13 (NL 1350)	3018.25	-0.254	3.58 × 10^−10^
6	14 (NL 1420)	3146.75	-0.208	2.93 × 10^−10^
7	15 (NL 1384)	2217.5	0.240	-3.39 × 10^−10^
8	16 (NL 1346)	2484	-0.009	1.31 × 10^−11^
9	17 (NL 1404)	2384.25	-0.330	4.66 × 10^−10^
10	18 (NL 1413)	2698.5	0.036	-5.11 × 10^−11^
11	19 (NL 1386)	2398.25	1	-1.41 × 10^−09^
12	2 (BL 4669)	2828.5	-0.873	1.23 × 10^−09^
13	20 (NL 1381)	2239.75	-0.082	1.16 × 10^−10^
14	3 (NL 1412)	2111.25	0.505	-7.13 × 10^−10^
15	4 (BL 4407)	2976.25	0.050	-7.16 × 10^−11^
16	5 (NL 1368)	3040.5	-0.172	2.44 × 10^−10^
17	6 (NL 1417)	2057	0.058	-8.20 × 10^−11^
18	7 (Bhrikuti)	3079.75	-0.653	9.22 × 10^−10^
19	8 (BL 4919)	2880.5	-0.165	2.33 × 10^−10^
20	9 (NL 1376)	2629.5	0.561	-7.92 × 10^−10^

PC 1 score revealed that NL 1386, NL 1179, NL 1376, NL 1412 are relatively unstable line with scores of 1, 0.627, 0.561, 0.505 respectively while PC 2 score shows that NL 1179, Gautam, BL 4669 with score of 9.24 × 10^−09^, 3.46 × 10^−09^, 1.23 × 10^−09^ are relatively unstable lines with regards to yield across both the test environments.

The interaction principal component of AMMI (1 & 2) with a yield of the 20 elite wheat lines are as follows as presented in Table 6.

### 3.2 GGE biplot analysis

#### 3.2.1 Which-won-where model

**Figure 3:**
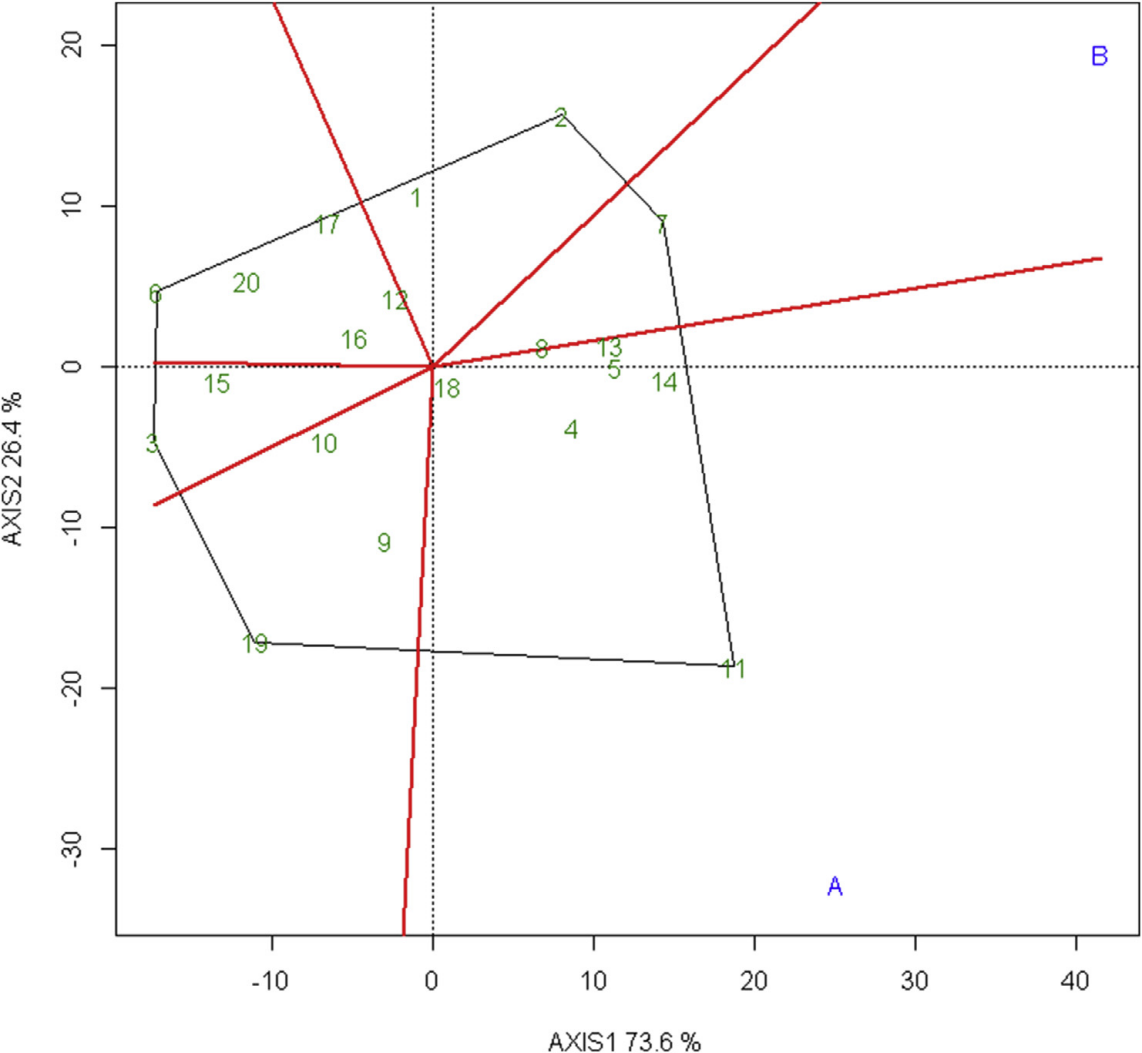
Polygon view of GGE biplot (which-won-where model) showing 20 elite wheat line in irrigated and terminal heat stressed environments.

The most effective and succinct way of summarizing the genotype and genotype-environment interaction of the dataset is the polygon-view of GGE biplot which visualizes the which-won-where pattern of a multi-environment dataset (Yan and Kang, 2003). The polygon is drawn by joining the markers located farthest from the origin such that all other markers are included within the polygon. The polygon view of this experiment as shown in Figure 3 revealed the 20 elite wheat lines fall under 6 sector and 2 test environment fall under 2 sectors in the polygon. The sector with irrigated environment consists of elite wheat BL 4407, NL 1368, NL 1179, and NL 1420; indicating these lines are responsive in this environment. The elite wheat NL 1179 vector is characterized by the longest distance from the origin and is the vertex line of this sector implies NL 1179 with specific adaptation in the irrigated environment but lower stability in the overall environment. Likewise, the sector with terminal heat-stressed environment consists of Bhirkuti; which is responsive in this environment. Furthermore, Bhirkuti vector is characterized by the longest distance from the origin and is the vertex line of this sector indicating this as the most responsive in terminal heat-stressed environment.

Thus, the which-won-where pattern of the trail revealed line NL 1179 as wining line in the irrigated environment while Bhirkuti as wining line in the heat-stressed environment.

In addition, the polygon view showed elite wheat NL 1413 near the origin of the biplot which means this line ranks the same in both test environments and is the most stable line. Also, elite wheat Gautam, BL 4669, NL 1412, NL 1417, NL 1376, NL 1387, NL 1369, NL 1384, NL 1346, NL 1404, NL 1386, NL 1381 are present in the sector with no test environment symbolizes these lines are poorly adapted to both the environments.

Similar to this research, Thungo et al. (2020) and Poudel et al. (2020b) also identified the high responsive genotype of wheat in heat-stressed environment using which -won-where model of GGE biplot; and noted the vertex genotype as wining genotype in the corresponding environment. Also, Neisse et al. (2018) were able to identify high-yielding and specifically adapted variety to a specific environment deploying which-won-where model. Similar, findings were mentioned by Gupta et al. (2017) and Kendal (2019).

#### 3.2.2 Mean vs. stability

**Figure 4:**
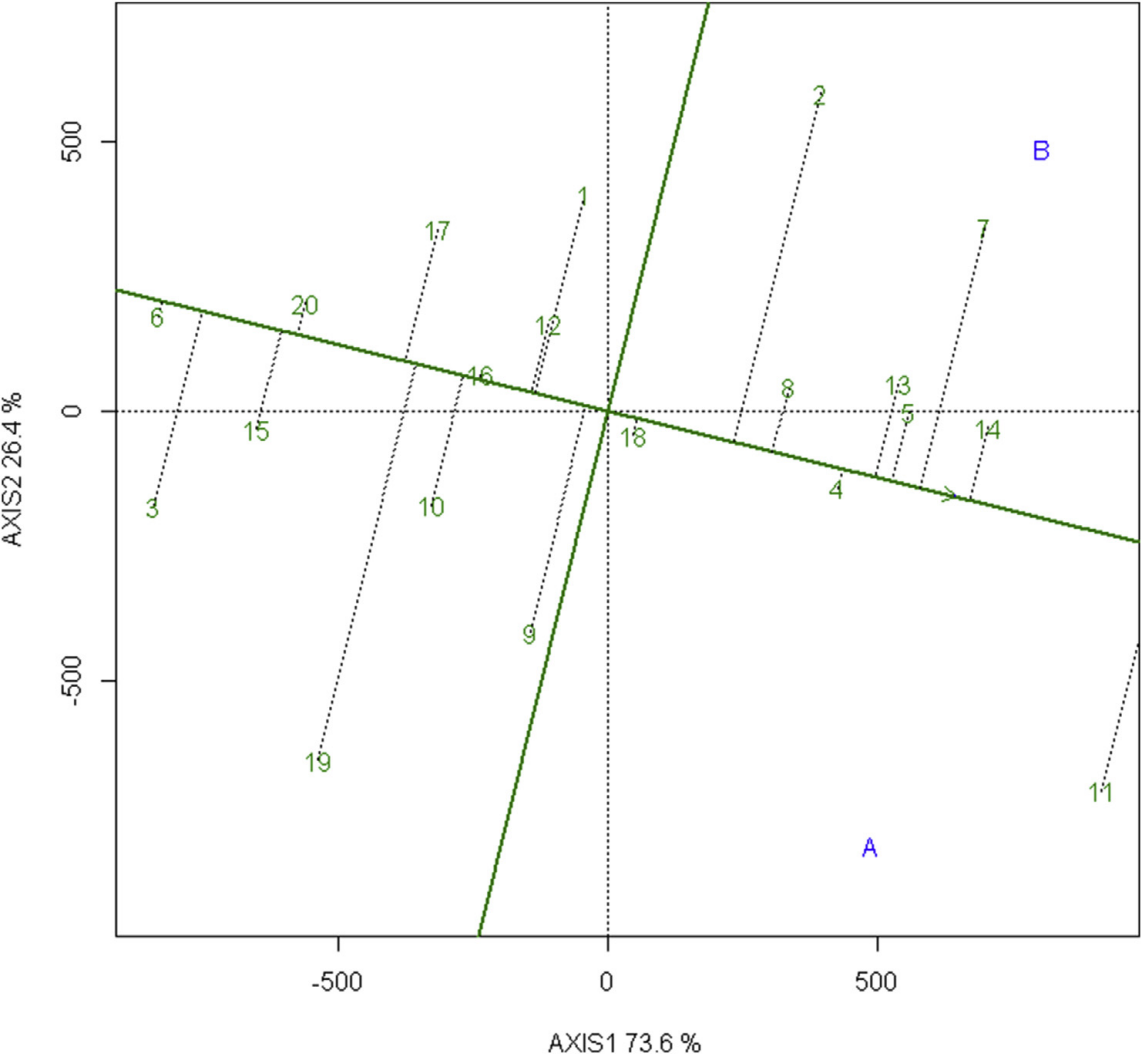
Mean vs. Stability view of GGE biplot showing the mean performance and stability of 20 elite wheat line in irrigated and terminal heat stressed environments.

When which–won-where pattern suggested wining elite wheat lines in the environments, there is a need to analyze the mean performance and stability of all the elite wheat lines to make selection decision. GGE biplot visualizes performance and stability graphically with the help of Average Environment Coordinates (AEC). AEC is the mean of first and second principal components scores of the test environments which is represented by the arrowhead in the Figure 4. The line passing through arrowhead and origin is AEC abscissa and the line perpendicular to it at origin is ordinate.

Length of Abscissa gives the yield of genotypes that is above-average and below-average yield if right and left of the origin respectively, and length of ordinate approximate the GEI associated with the genotype that is more length corresponds higher variability and lower stability and vice-versa.

Figure 4 shows elite wheat BL 4407, NL 1368, BL 4919, NL 1350, NL 1420, NL 1413 are above-average yielders with more stability whereas BL 4669, Bhrikuti, NL1179 are above-average yielders but with lower stability. Moreover, NL 1417, NL 1369, NL 1384, NL 1346, NL 1381 are stable but are below-average yielders and Gautam, NL 1412, NL 1376, NL 1387, NL 1404, NL1386 are both less stable and below-average yielders.

Ideal lines have the highest yield and absolute stability lying in the arrowhead and the distance of other lines measures the desirability of lines. Figure 4 show the desirability of these lines, NL 1420 which lies closest to the AEC is most desirable line. This desirability gives the ranks of the line in order NL 1420 followed by Bhrikuti, NL 1368, NL 1350, BL 4407, and NL 1179.

Neisse et al. (2018) also evaluated genotypes using Mean vs stability GGE biplot and identified high-yielding and stable genotype deploying Average Environment Coordinate (AEC). Similar observation of stable genotypes was observed using Average Environment (tester) Coordinate methods by Yan (2001), Yan and Hunt (2001), Singh et al. (2019) and Poudel et al. (2020b).

#### 3.2.3 Ranking elite wheat lines (genotypes)

The ideal line which is practically not possible lies in the arrowhead. In order to rank the lines, two coordinate axis are drawn- a line joining arrowhead and origin: first axis and the line perpendicular to it at the origin: second axis. Then by observing concentric circles along the arrowhead we can further rank the lines as per inclusion in the circles and the distance from arrowhead in the ordinate.

**Figure 5:**
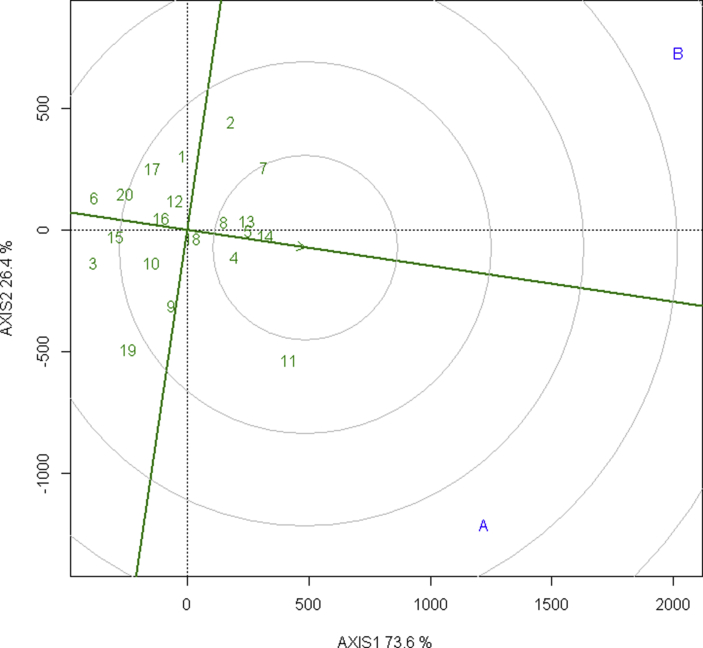
GGE biplot showing the ranking of 20 elite wheat line about the ideal line in irrigated and terminal heat stressed environments.

The elite wheat NL 1420 is very close to the ideal line which can be used as a reference in the evaluation of the lines. This is followed by NL 1368, NL 1350, BL 4407, BL 4919 in the rank of desirable genotypes which could be used for further testing in the heat stress and non-stress environments as shown in Figure 5. The general ranking from the biplot is as follows:

NL 1420 > NL 1368 > NL 1350 > BL 4407> BL 4919> Bhrikuti > NL 1179> BL 4669 > NL 1413 >NL 1376 > NL 1369 > Gautam > NL 1346 > NL 1387 > NL 1404 > NL 1381 > NL 1384 > NL 1386 > NL 1412 > NL 1417.

The method of identifying ideal genotype as high-yielding and stable across the test environments, is in accordance to Yan and Kang (2003), and Yan and Tinker (2006). The desirability of genotype based on the closer location to ideal genotype is similar to Yan (2001). Similar, findings and method was observed in Akther et al. (2015).

The comparison of biplot ranking and mean yield ranking of the genotypes in the combined environment (terminal heat stress and irrigated environment) is given in Table 7.

**Table 7:** Comparison of the rank of 20 elite wheat lines based on mean yield and biplot ranking.

Genotype Rank	Mean Yield Ranking	Biplot Ranking
1	NL1179	NL1420
2	NL1420	NL1368
3	Bhirkuti	NL1350
4	NL1368	BL4407
5	NL1350	BL4919
6	BL4407	Bhirkuti
7	BL4919	NL1179
8	BL4669	BL4669
9	NL1413	NL1413
10	NL1376	NL1376
11	Gautam	NL1369
12	NL1369	Gautam
13	NL1346	NL1346
14	NL1387	NL1387
15	NL1386	NL1404
16	NL1404	NL1381
17	NL1381	NL1384
18	NL1384	NL1386
19	NL1412	NL1412
20	NL1417	NL1417

#### 3.2.4 Discriminativeness vs. representativeness

The environment with no discriminating ability gives no information of lines that is useless and the environment that is not representative is useless as well as misleading.

**Figure 6:**
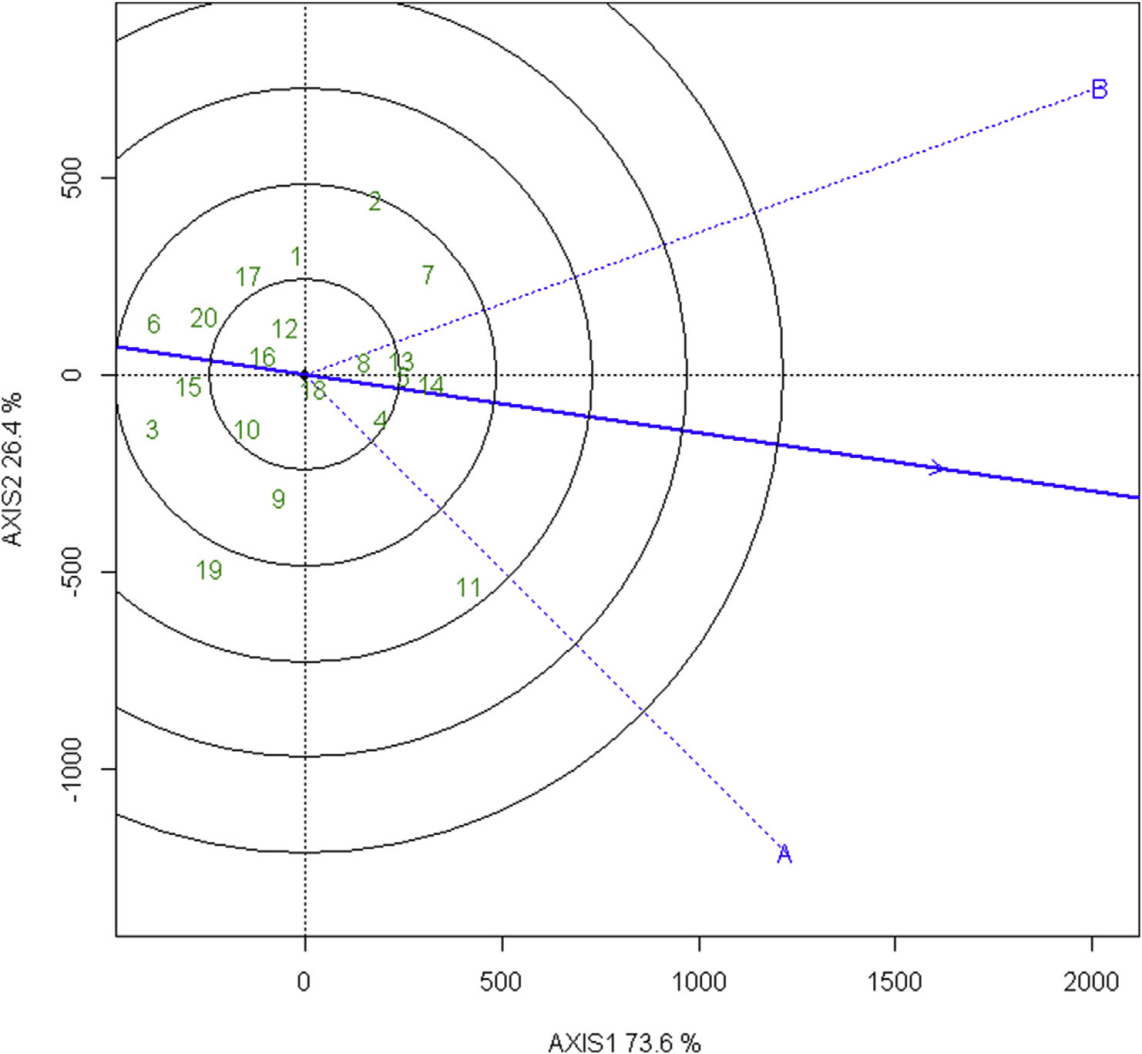
Discriminativeness vs. representativeness view of GGE biplot showing 20 elite wheat lines in irrigated and terminal heat stressed environments.

The GGE biplot uses the vector of the environment to measure discriminativeness that is longer the length of the environment vector higher is the standard deviation within the environment indicating higher discriminating ability. The heat stress environment vector has comparatively more length ensuring it has a higher discriminating ability as shown in Figure 6. Furthermore, the cosine of the angle between the environments gives the interrelationship between the environments that is angled just less than 90 ° shows a positive but low correlation coefficient between terminal heat stress and irrigated environment. Since the angle is large the environments are not redundant.

Representativeness is a measure of environment similar to the AEC ranking of genotypes. The desirability of the environment is not seen because of the use of few environments. But, both the environment vector inscribe somewhat equal angles to the average environment coordinate symbolizes similar representativeness of irrigated and terminal heat stressed environment as shown in Figure 6.

The discriminativeness vs representativeness view of GGE biplot allows evaluation of environment that is advantageous over AMMI biplot (Yan et al., 2007; Aktas, 2016). Similarly, it also helps to find the environment capable of selecting superior genotypes in an efficient way. Discriminative vs. Representative GGE biplot has already been used by Philipo et al. (2021), Kendal (2019), and Gupta et al. (2017) to compare the discriminating ability and desirability of the environments.

## 4 Conclusion

This study indicated that genotype, environment, and their interaction have a significant effect on the yield stability and 100% of the interaction effect was explained by PC 1 as per the AMMI model. Further analysis of stability through GGE biplot concluded elite wheat line NL 1179 was specifically adapted to the irrigated environment whereas Bhirkuti was specifically adapted to the terminal heat stressed environment via. which-won-where model however were among the least stable lines along with BL 4669 and NL 1386. The study of the mean vs stability and ranking of the line in the GGE biplot revealed, elite wheat line NL 1420 along with NL 1368, NL 1350, BL 4919 as above-average yielder lines and more desirable lines close to the ‘ideal line’. In this experiment, NL 1413 was found to be the most stable line across both the test environment as per AMMI biplot, which-won-where GGE biplot and mean vs stability GGE biplot. The terminal heat-stressed environment had a slightly higher discriminating ability than irrigated environment and comparatively equal representativeness. All in all, for breeding programs NL 1420 can be used as a high yielding line which is stable too, and for farmers NL 1179 and NL 1350 can be used for high yield with adaptability in irrigated and heat-stressed environments, respectively. These lines NL 1413, NL 1179, and NL 1350 need to be further tested in heat-stressed environment to ensure their performance over years.

## Declarations

### Author contribution statement

Bishwas K. C.: Conceived and designed the experiments; Performed the experiments; Analyzed and interpreted the data; Contributed reagents, materials, analysis tools or data; Wrote the paper.

Mukti Ram Poudel: Conceived and designed the experiments; Analyzed and interpreted the data; Contributed reagents, materials, analysis tools or data; Wrote the paper.

Dipendra Regmi: Performed the experiments; Analyzed and interpreted the data; Wrote the paper.

### Funding statement

This research did not receive any specific grant from funding agencies in the public, commercial, or not-for-profit sectors.

### Data availability statement

Data included in article/supplementary material/referenced in article.

### Declaration of interests statement

The authors declare no conflict of interest.

### Additional information

No additional information is available for this paper.
